# Trans‐reflective color filters with brilliant colors by integrating organic dyes with photonic crystals

**DOI:** 10.1002/smo2.70037

**Published:** 2026-01-30

**Authors:** Shi Li, Yong Qi, Wenbin Niu, Suli Wu, Bingtao Tang, Wei Ma, Shufen Zhang

**Affiliations:** ^1^ State Key Laboratory of Fine Chemicals, Frontier Science Center for Smart Materials Dalian University of Technology Dalian China

**Keywords:** dyes, photonic crystals, stability, trans‐reflective color filters

## Abstract

Color filters are essential components for optical modulation. However, conventional filters are restricted to operating exclusively in either reflective or transmissive mode. Furthermore, they suffer from limited UV and thermal stability, low color purity, and exhibit identical coloration on both surfaces. Herein, we propose a novel design strategy for trans‐reflective color filters by integrating the absorptive properties of dye‐doped polysulfone (PSU) with the diffractive capabilities of photonic crystals. This composite filter achieved broad‐spectrum transmission with deep color outputs—yellow (0.410, 0.510), magenta (0.446, 0.231), and cyan (0.201, 0.425)—closely aligned with standard color space coordinates. By tuning the refractive index of CeO_2_@SiO_2_ nanoparticles to match dye‐based PSU matrix, the transmittance of filters exceeded 70%. Moreover, dye‐mediated absorption reduces the scattering light, thereby enhancing reflection color purity (full width at half maxima (FWHM) = 25 nm) and producing vibrant blue, green, and red hues. The incorporation of UV‐absorbing CeO_2_@SiO_2_ nanoparticles effectively mitigated dye photodegradation, yielding exceptional UV stability (Δ*T* < 2% under prolonged UV exposure). The filters also exhibited outstanding thermal stability (Δ*T* < 1% after 30 min heat treatment at 230°C). This work establishes a robust materials design framework for multifunctional optical filters, advancing the development of high‐fidelity dual‐mode color systems for next‐generation display technologies.

## Graphical abstract

A novel trans‐reflective color filter design achieves high color purity, >70% transmittance, and exceptional UV/thermal stability by integrating dye‐doped polysulfone with photonic crystals, enabling advanced dual‐mode displays.

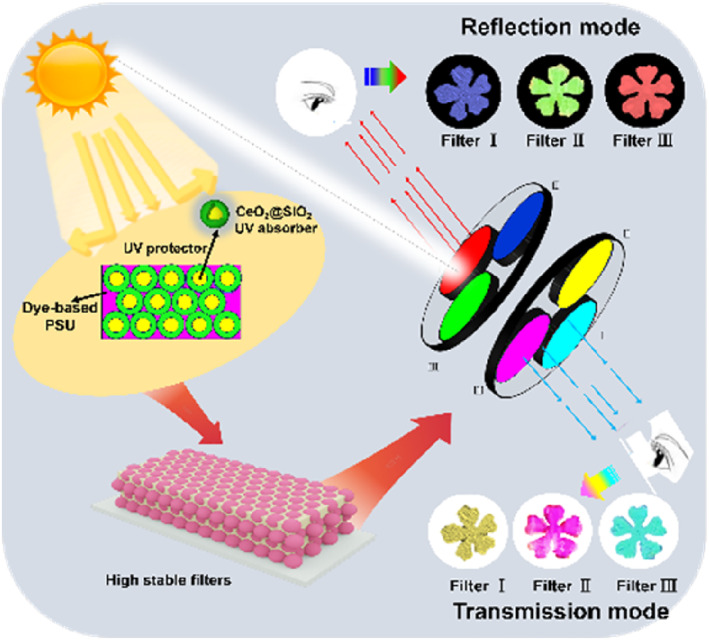

## 1 INTRODUCTION

With advancements in optoelectronic devices, color filters have become essential for light modulation, with widespread applications in laser experiments, display technologies,^[1]^ spectral analysis,^[2]^ and optical measurement systems. Traditional dye‐ and pigment‐based color filters have long been prevalent in various fields due to their high light transmission and cost‐effectiveness. The fundamental principle of these filters is based on the electronic structure of dye molecules, particularly the energy‐level differences between molecular orbitals, which determine the absorbed wavelengths. Selectively absorb specific wavelengths of light, while transmitting or reflecting the unabsorbed portions to produce color. They are widely used in display technologies, optical sensors, and camera filters.[^3^, ^4^, ^5^, ^6^, ^7^, ^8^, ^9^] However, several challenges remain, including difficulties in material selection, limited UV stability and thermal stability, suboptimal peak transmission efficiency, and challenges in modulating the steepness of the absorption edge. In recent years, structural color filters leveraging the optical resonant properties of nanostructures have advanced rapidly and gained attention for their precise spectral selectivity and stability. For example, high‐purity reflective color filters have been achieved by combining Fabry‐Pérot microcavity structures with organic dye films.[^10^, ^11^] Similarly, one‐dimensional (1D) nanogratings and plasmonic resonance mechanisms have enabled high‐efficiency transmissive color filters.[^12^, ^13^, ^14^, ^15^, ^16^] However, existing structural filters generally operate exclusively in either transmission or reflection mode.

Trans‐reflective color filters, which can function in both reflective and transmissive modes, have attracted growing attention. Recently, a novel design based on asymmetric microcavity structures (Thin‐Ag–HATCN–Thick‐Ag layers) can produce additive RGB (red, green, blue) colors in transmission and subtractive CMY (cyan, magenta, yellow) colors in reflection.[^12^, ^17^, ^18^, ^19^, ^20^] Despite this progress, such filters still suffer from low color purity, and their fabrication relies on a limited range of high‐refractive‐index (RI) materials (*n* > 3), indicating the need for further innovation. Photonic crystal (PC) demonstrates unique application value in the field of optical devices due to their advantages of simple preparation process, strong structural designability, and significant tunability of photonic bandgaps. Their periodic structure endows the material with dual‐effect optical control properties: Bragg diffraction enables high‐precision reflective filtering,[^21^, ^22^, ^23^] while photonic localization effects govern transmissive spectral modulation. However, single‐photon crystals often exhibit light scattering in non‐bandgap regions due to refractive index differences in the dielectric material, resulting in a significant decrease in the purity of the transmitted spectrum and, consequently, a severe reduction in the color saturation and purity of these devices.[^20^, ^21^, ^22^, ^23^, ^24^] Dyes, with their specific wavelength‐selective absorption properties, can effectively suppress stray light generated by structural color while achieving transmissive filtering, thereby enhancing spectral purity.^[10]^ Based on the synergistic regulation of dye absorption and photonic bandgap, this study innovatively proposes a dye‐photonic crystal composite structure design scheme: the molecular absorption properties of dyes compensate for light scattering losses in the non‐bandgap regions of PCs, while leveraging the wide‐spectrum control capability of PCs to expand the dye's transmission filtering spectral band. This results in a composite system capable of producing significant spectral superposition effects, enhancing both the purity of the PC's reflected light and the purity of the transmitted light.

**SCHEME 1:**
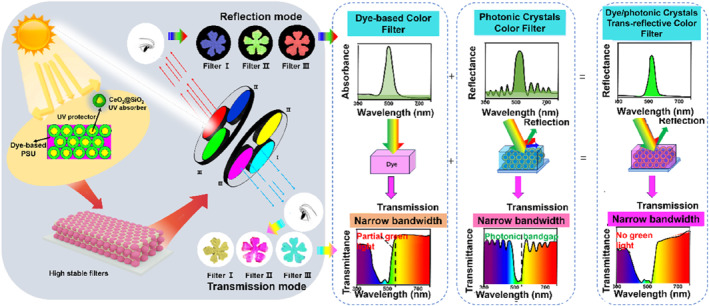
Design mechanism and application of vibrant dye/photonic crystal trans‐reflective color filters.

In this work, we fabricated novel trans‐reflective color filters by integrating yellow, magenta, and cyan dyes with the photonic bandgap located in the blue band at the maximum absorption wavelength of the dye (Scheme 1). Core–shell spheres composed of cerium dioxide (CeO_2_) and silicon dioxide (SiO_2_) were precisely engineered, with a refractive index closely matching that of the dye‐doped polysulfone (PSU) matrix. These spheres were employed to construct both PC structures and dye–PC composite filters. We also conducted spectral analysis and simulations using the finite‐difference time‐domain (FDTD) method to study the interaction between the photonic bandgap and dye absorption, and their combined effect on reflection and transmission. Based on these results, the developed trans‐reflective filters were applied to light modulation and patterning devices, demonstrating high color saturation, along with excellent thermal stability and UV stability. This approach simplifies the design of high‐performance trans‐reflective color filters, offering new opportunities for applications across diverse optoelectronic fields.

## 2 RESULTS AND DISCUSSION

### 2.1 Design and preparation of CeO_2_@SiO_2_ core‐shell microsphere

**FIGURE 1:**
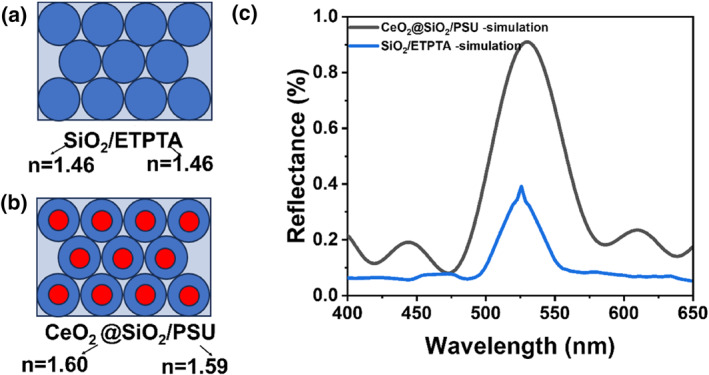
Spectral simulations of photonic crystals with different structures: (a) Schematic of SiO_2_‐ETPTA ordered hexagonal arrays; (b) Schematic of CeO_2_@SiO_2_‐PSU ordered hexagonal arrays; (c) Simulated reflectance spectras of SiO_2_‐ETPTA (n(SiO_2_) = 1.46, D(SiO_2_) = 220 nm) and CeO_2_@SiO_2_‐PSU (n(CeO_2_) = 2.20, D(CeO_2_) = 110 nm; n(SiO_2_) = 1.46, D(SiO_2_) = 90 nm) (*ϕ* = 0.74). ETPTA, poly(ethyl trimethylolpropane triacrylate); PSU, polysulfone.

Based on prior literature,^[24]^ PC composite films utilizing poly(ethyl trimethylolpropane triacrylate) (ETPTA) as the matrix material exhibit high transparency (Figure 1a) due to the refractive index matching between ETPTA and SiO_2_. However, the insufficient refractive index contrast (Δ*n* = 0.02) between SiO_2_ and ETPTA significantly limits structural color intensity. Increasing the PC film thickness to enhance structural color induces defects such as particle absence, dislocations, and cracks,^[22]^ consequently reducing transmittance and compromising suitability for high‐purity color filter applications. To address this limitation, finite element method (FEM) simulations revealed that core‐shell microspheres with a high‐RI core (*n* > 2.0) and a low‐RI shell can generate stronger Bragg reflection while maintaining high transparency at equivalent film thicknesses, provided the effective refractive index of the core‐shell structure matches that of the surrounding matrix.^[24]^ Consequently, this work employs a core‐shell design strategy. CeO_2_ was selected as the core material due to its high refractive index (*n* = 2.20) and inherent catalytic activity. SiO_2_, possessing no catalytic properties and a lower refractive index (*n* = 1.46), serves as the shell material, forming a CeO_2_@SiO_2_ core‐shell microsphere system (Figure 1b). FDTD simulations comparing the reflectance spectra of SiO_2_‐ETPTA and CeO_2_@SiO_2_‐polysulfone (PSU) composites (Figure 1c) demonstrate that the CeO_2_@SiO_2_‐PSU system achieves a significantly higher reflectance of approximately 90% under identical conditions, compared to ∼35% for the SiO_2_‐ETPTA film of equivalent thickness. The CeO_2_ cores were controllably synthesized via an alcohol‐thermal method, with particle size modulated by adjusting the amount of cerium(III) nitrate hexahydrate. The SiO_2_ shell (thickness: 40–90 nm) was subsequently deposited onto the CeO_2_ cores using a sol‐gel process, enabling dynamic refractive index matching with the polymer matrix (Δ*n* < 0.05).

### 2.2 Design and preparation of DPCCFs

**FIGURE 2:**
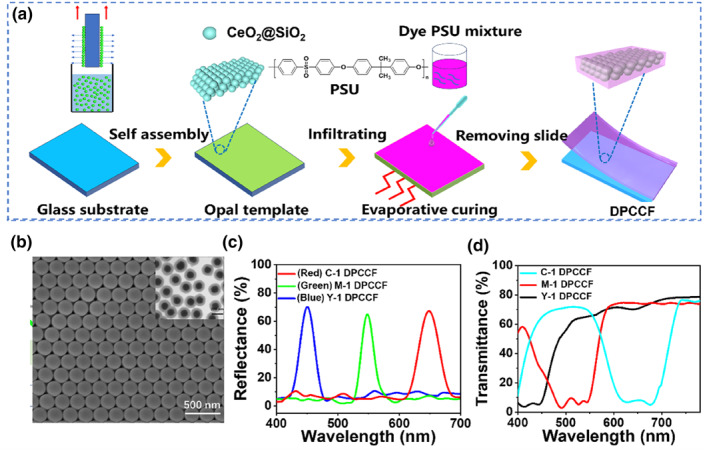
Construction of the DPCCF. (a) Preparation route of DPCCF based on index‐matched CeO_2_@SiO_2_ nanospheres in a PSU matrix; (b) SEM and TEM images of 215 nm CeO_2_@SiO_2_ core–shell nanospheres; (c) Reflection spectra of Y‐1 DPCCF, M‐1 DPCCF, and C‐1 DPCCF with PC thickness at 26 μm; (d) Transmission spectra of Y‐1 DPCCF, M‐1 DPCCF, and C‐1 DPCCF with PC thickness at 26 μm. DPCCF, dye/photonic crystal trans‐reflective color filter; PC, photonic crystal; PSU, polysulfone; SEM, scanning electron microscopy; TEM, transmission electron microscope.

Three organic dyes (Y‐1, M‐1, and C‐1) shown Figure S2 in Supporting Information S1, previously verified in our studies for their excellent solubility in propylene glycol methyl ether acetate (PGMEA)—a solvent commonly used in industrial color filter production—were selected for the dye/photonic crystal trans‐reflective color filters (DPCCFs).[^25^, ^26^, ^27^] A schematic view of the high‐purity trans‐reflective color filters is shown in Figure 2a. To construct the PC structure, we chose CeO_2_@SiO_2_ nanospheres due to its excellent UV‐blocking properties and adjustable refractive index.^[28]^ The photonic bandgap is located in the blue band at the maximum absorption wavelength of the dye. Details of the CeO_2_@SiO_2_ preparation are provided in Table S1. The uniformity of the nanospheres makes them highly suitable for PC construction.

Furthermore, the low‐magnification cross‐sectional scanning electron microscopy (SEM) image in Figure 2b reveals a long‐range, close‐packed array of 215 nm CeO_2_@SiO_2_ core–shell nanospheres throughout the composite films, as confirmed by the transmission electron microscope (TEM)  image at the top right corner in Figure 2b. The thickness of PC was set to 26 μm, ensuring both high reflectance and transmittance (∼78%) of the composite films, as reported in previous study.^[24]^ The opal templates were infused with PSU polymers whose refractive index closely matched that of the core–shell spheres, resulting in the formation of DPCCFs. Dyes were optimized at same concentrations with the DPCCFs to create dye‐based composite filters (DCFs). For comparison, CeO_2_@SiO_2_ opals used in DPCCF preparation were also employed as templates and filled with PSU dispersion to produce PC composite filters (Figures S2–S9), the final reflection and transmission spectra of DPCCFs are shown in Figure 2c,d.

### 2.3 Mechanisms of color formation in DPCCFs

**FIGURE 3:**
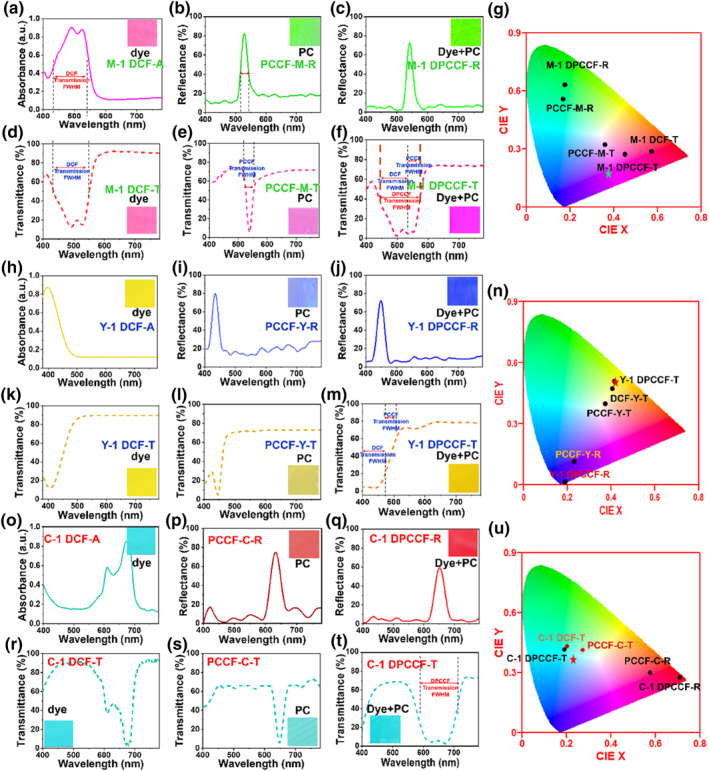
Spectra property of dye, PC, and DPCCF. (a, h, o) Absorption spectra of M‐1 DCF, Y‐1 DCF, C‐1 DCF; (b, c, i, j, p, q) Reflection spectra of PCCF‐M, PCCF‐Y, PCCF‐C, M‐1 DPCCF, Y‐1 DPCCF, and C‐1 DPCCF; (d–f, k–m, r–t) Transmission spectra of M‐1 DCF, Y‐1 DCF, C‐1 DCF, PCCF‐M, PCCF‐Y, PCCF‐C, M‐1 DPCCF, Y‐1 DPCCF, and C‐1 DPCCF; (g, n, u) CIE 1931 chromaticity diagram of the reflection color and transmission color of the filters (The red five‐pointed star in the CIE diagram is a triangle showing the pure CMY value *c* obtained from the transformation matrix). CMY, cyan, magenta, yellow; DCF, dye‐based composite filter; DPCCF, dye/photonic crystal trans‐reflective color filter; PC, photonic crystal.

To investigate the individual contributions of dyes and PCs on the optical performance of trans‐reflective color filters, the spectral properties of M‐1 DCF, PCCF‐M, and M‐1 DPCCF were systematically measured, as shown in Figure 3.

The M‐1 DCF, composed of perylene diimides dye M‐1 and PSU, exhibits an absorption spectrum with maximum absorption wavelength between 482 and 530 nm, and a nearly flat baseline between 580 and 780 nm, indicating negligible absorption (Figure 3a). The corresponding color block in Figure 3a displays a vibrant magenta hue. In contrast, the PC composite filter (PCCF‐M), which integrates a CeO_2_@SiO_2_ nanosphere (215 nm diameter) PC layer within a PSU substrate, shows a reflection peak at 542 nm (Figure 3b). However, due to light scattering by the microspheres, reflectance remains above 20% in the non‐reflective region (580–780 nm), diminishing reflection color brightness and resulting in a dull color block. The M‐1 DPCCF, which combines dye M‐1 with a PC structure, shows a reflection peak at 550 nm, and the reflectance of DPCCF‐M in the non‐reflective region (580–780 nm) is below 5%. This low reflectance could be attributed to both absorption in the M‐1 dye‐doped PSU and a slight increase in its refractive index—the latter shifting the composite's optical properties closer to CeO_2_@SiO_2_ than pure PSU. Consequently, the baseline in the 580–780 nm range becomes flatter, and the reflection peak turns sharper, enhancing color purity of the composite filter at the cost of slightly reduced reflectance intensity (Figure 3c). Chromaticity coordinates further highlight the enhancement in color purity achieved by incorporating dye M‐1 into PC structure. PCCF‐M reflects light with chromaticity coordinates of (0.173, 0.562), while M‐1 DPCCF reflects at (0.186, 0.635), closer to the standard green point (0.150, 0.600) used in liquid crystal displays (Figure 3g). These results demonstrate that incorporation of dye M‐1 effectively reduces the scattered light generated within the structure and suppresses reflections in the range of non‐target colors, thereby enhancing the purity of the reflection color.

The transmission spectrum of the M‐1 DCF is complementary to its absorption spectrum, resulting in a magenta color block (Figure 3d). The color block magenta in Figure 3e is darker than the M‐1 DCF transmission color, and the reflection band in PCCF‐M is narrow (full width at half maxima (FWHM) = 30 nm), which leads to a large amount of unblocked light of green wavelengths remaining in the transmission spectrum, and finally the purity of the transmission color is reduced, and thus the magenta color is dull. In contrast, the M‐1 DPCCF composite filter shows a transmission spectrum as shown in Figure 3f, which maintains a maximum transmittance of ∼70%, and its transmission spectrum is a superposition of the dye transmission spectrum and the PC transmission spectrum, with the dye partially correcting the light scattering phenomenon in the non‐transmission spectral region of the PC, and the transmission spectrum of the M‐1 DPCCF has a band‐resistant FWHM of ∼80 nm, which is much higher than that of a single PCCF‐M (30 nm) and M‐1 DCF (60 nm). As a result, the M‐1 DPCCF forms a deeper magenta color.^[11]^ The CIE 1931 chromaticity coordinates of the transmitted light colors of the three filters, M‐1 DCF, PCCF‐M, and M‐1 DPCCF, are shown in Figure 3g. PCCF‐M reflects light with chromaticity coordinates of (0.173, 0.562), while M‐1 DPCCF reflects at (0.186, 0.635), closer to the standard green point (0.150, 0.600)^[28]^ used in liquid crystal displays (Figure 3g). The result is due to the synergistic action of PC reflection and dye absorption that prevents light in the green band from transmitting through the film, and therefore the resulting transmitted light magenta has a higher color purity, and thus the film has a darker magenta color than that of PCCF‐M. This improvement is attributed to the combined effects of dye absorption and PC structuring, which effectively minimize light scattering and enhance overall optical performance.

The increase in color purity of both reflection and transmission, resulting from the combination of dyes with corresponding PCs, is also observed in yellow and cyan composite filters. Figure 3h shows the absorption spectrum of the azo dye‐based filter Y‐1 DCF, while Figure 3i,j present the reflection spectrum of single PC filter PCCF‐Y (constructed by integrating a 175 nm CeO_2_@SiO_2_ PC layer with PSU) and the composite filter Y‐1 DPCCF (incorporating Y‐1 with CeO_2_@SiO_2_/PSU), respectively. In Y‐1 DPCCF, reflectance in the 480–780 nm range is reduced to ∼5%, resulting in a more vibrant blue color in the reflection color block compared to PCCF‐Y. Furthermore, the broader transmission spectrum of Y‐1 DPCCF (the FWHM of the transmission spectral band rejection: ∼80 nm, Figure 3m) reflects a combination of the spectral features of Y‐1 DCF (∼60 nm, Figure 3k) and PCCF‐Y (∼30 nm, Figure 3l), yielding a bright yellow color with high transmittance (∼75%) and increased purity. The chromaticity coordinates of Y‐1 DPCCF—(0.190, 0.010) for reflection and (0.410, 0.510) for transmission—lie closer to the standard yellow coordinates (0.416, 0.515) (Figure 3n),^[28]^ indicating superior color purity compared to both Y‐1 DCF and PCCF‐Y.

A similar enhancement is observed in the cyan system based on the phthalocyanine dye C‐1. Figure 3o presents the absorption spectrum of C‐1 DCF, while Figure 3p,q showing the corresponding reflection spectrum of PCCF‐C and C‐1 DPCCF, respectively. In C‐1 DPCCF, reflectance in the non‐reflective regions (400–600 nm and 700–780 nm) drops to ∼5%, resulting in a more vibrant red hue in the reflection color block compared to PCCF‐C. The transmission spectrum of C‐1 DPCCF shows a broadened spectral filtering band (the FWHM of the transmission spectral band rejection: ∼90 nm), demonstrating a synergistic effect between the dye and PC. This leads to a more brilliant cyan coloration with a maximum transmittance of ∼70% (Figure 3r–t). Chromaticity coordinates further confirm the enhanced optical performance of C‐1 DPCCF (Figure 3u). The reflected light of C‐1 DPCCF corresponds to coordinates (0.712, 0.281) in the red region, while the transmitted light is located at (0.201, 0.425) in the cyan region. The coordinate lie closer to the standard cyan coordinates (0.254, 0.383), indicating higher color purity and deep transmission color (cyan) compared to the individual components.

In summary, the integration of dye‐based PSU with PCs not only significantly enhances the purity of both reflected and transmission colors in the filters, but also optimizes their spectral characteristics through synergistic effects, which leads to superior color purity and overall optical performance.

### 2.4 Simulation and analysis of optical properties of DPCCFs

**FIGURE 4:**
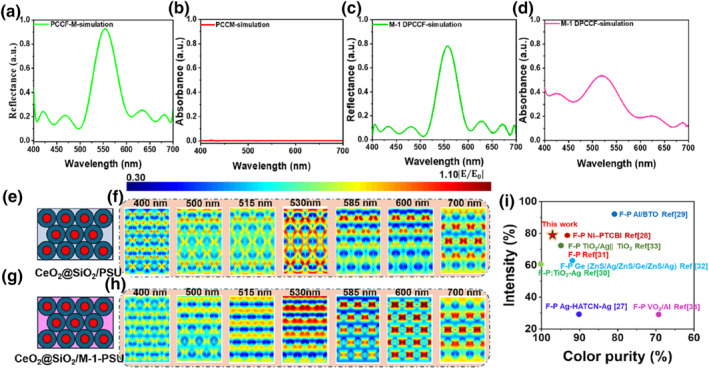
Simulation results and summary of the optical properties of PCCF‐M and M‐1 DPCCFs: (a, c) Simulated reflection spectra of PCCF‐M and M‐1 DPCCFs; (b, d) Simulated absorption spectra of PCCF‐M and M‐1 DPCCFs; (e, g) Structural diagrams of PCCF‐M and M‐1 DPCCFs; (f, h) Simulated electric field distribution of the proposed PCCF‐M and M‐1 DPCCFs at different wavelengths (400, 500, 525, 535, 585, and 700 nm), the electric field distribution of the internal structure of the proposed PCCF‐M and M‐1 DPCCFs; (i) The summary of color purity and light intensity of trans‐reflective color filter based on nanostructure (red star represents our work). DPCCFs, dye/photonic crystal trans‐reflective color filters.

As previously discussed, the synergistic interaction between dyes and PCs enhances the contrast and purity of both reflection and transmission color in composite filters. To further investigate this, the optical properties of M‐1 DCF, PCCF‐M, and M‐1 DPCCF were simulated using the FDTD method, as illustrated in Figure 4. The structural configurations of PCCF‐M (CeO_2_@SiO_2_/PSU) and M‐1 DPCCF (CeO_2_@SiO_2_/M‐1‐PSU) are depicted in Figure 4e,g. The simulated reflection spectrum (Figure 4a) shows a maximum reflectance wavelength of 549 nm for PCCF‐M, with 19% reflectance in the non‐reflective region (600–780 nm). In contrast, M‐1 DPCCF exhibits a slightly shifted maximum reflectance wavelength at 554 nm (Figure 4c) and a significantly reduced reflectance of only 6% in the same region, resulting in a narrower and sharper reflection peak. Chromaticity coordinates for PCCF‐M and M‐1 DPCCF are (0.253, 0.468) and (0.236, 0.527), respectively (Figure S10), confirming that the dye improves reflection color purity. These simulation results are consistent with the experimental data discussed earlier.

To further analyze the interaction mechanism, the absorption spectra and electric field distributions of various structures were simulated using the FDTD method. The refractive index (*n*) and extinction coefficient (*k*) of M‐1 DCF (0.90 wt% dye concentration) were obtained via ellipsometry (Figure S1), and subsequently used to calculate the absorption spectra for M‐1 DCF (Figure S11) and M‐1 DPCCF (Figure 4d). M‐1 DPCCF retains the absorption characteristics of M‐1 DCF but shows enhanced absorption at its maximum wavelength (530 nm) and a narrower absorption band. This is attributed to resonance effects from the PC structure, which amplifies M‐1 optical absorption at its maximum absorption wavelength. PCCF‐M, by contrast, exhibits negligible visible‐light absorption (Figure 4b).

Simulated electric field modulus distributions for PCCF‐M and M‐1 DPCCF across visible wavelengths (400, 500, 515, 530, 585, 600, and 780 nm) are shown in Figure 4f,h. Electric field strength is represented via a color scale corresponding to photon energy intensity.^[29]^ For PCCF‐M (Figure 4a), the maximum reflection occurs at ∼544 nm, with light scattering observed in other visible regions (585–700 nm). This phenomenon is clearly evident in the electric field distribution for PCCF‐M shown in Figure 4f. In high‐scattering regions (>585 nm), microsphere surfaces exhibit intense red coloration with field strength around 1.00 [*E*/*E*_0_], with energy coloration shifting from red to blue along the vertical axis, while in low‐scattering regions (<515 nm), the surfaces display orange‐red coloration with field strength of approximately 0.88 [*E*/*E*_0_]. At 530 nm, which is closest to the maximum reflection wavelength, field strength reaches 1.08 [*E*/*E*_0_] with appearance of high‐energy red coloration, and the energy coloration had no change along the vertical axis, which is attributed to the negligible visible‐light absorption of PCCF‐M. M‐1 DPCCF (Figure 4h) shows a similar electric field distribution to PCCF‐M, with maximum reflection wavelength at ∼550 nm (Figure 4c) and light scattering in other visible regions. In high‐scattering regions (>585 nm), it presents high‐energy red coloration with a field strength of around 0.97 [*E*/*E*_0_]; in low‐scattering regions (<515 nm), an orange‐red coloration appears with a field strength of approximately 0.83 [*E*/*E*_0_]. Notably, at 530 nm, closest to the maximum reflection wavelength, the surface field strength of M‐1 DPCCF fabricated by CeO_2_@SiO_2_/M‐1‐PSU microspheres are slightly reduced to 1.03 [*E*/*E*_0_], caused by M‐1 dye absorption dissipating part of the energy. Additionally, at 530 nm, electric field intensity of M‐1 DPCCF drops to 0.72 [*E*/*E*_0_], with energy coloration shifting from red to green along the vertical axis—evidence of increased dye absorption along the longitudinal structure, this result is consistent with above absorbance spectrum of DPCCF‐M.

The findings above confirm that incorporating dye into PC structure can effectively suppress light reflection of PCs and improve transmission performance. To date, trans‐reflective color filters with such high reflection color purity and transmittance up to 70% are rarely reported, as summarized in Figure 4i. The high color purity and brightness of these filters, coupled with their potential for downscaling to sizes compatible with modern CMOS imagers, make them promising for fine‐tuned applications in advanced optical instrumentation.[^30^, ^31^, ^32^, ^33^, ^34^, ^35^, ^36^, ^37^]

### 2.5 Light and thermal stability of DPCCFs

**FIGURE 5:**
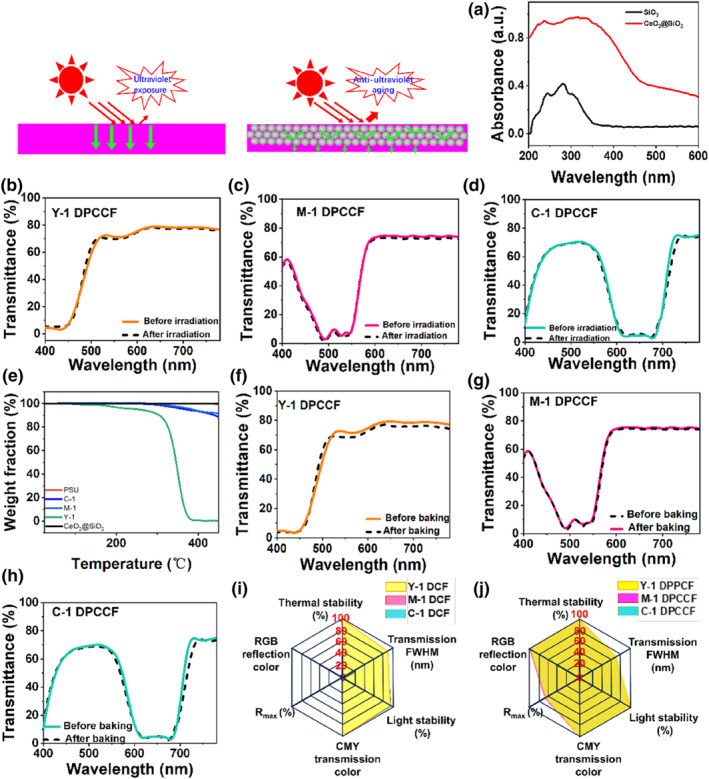
Stability of three DPCCFs. (a) Absorbance spectrum of SiO_2_ and CeO_2_@SiO_2_ nanospheres in ethanol solution (0.001 wt%); (b–d) Transmission spectrum of Y‐1 DPCCF, M‐1 DPCCF, C‐1 DPCCF before and after irradiation, respectively; (e) TGA curves of PSU, organic dyes and CeO_2_@SiO_2_ nanospheres; (f–h) Transmission spectrum of Y‐1 DPCCF, M‐1 DPCCF and C‐1 DPCCF before and after baking, respectively; (i, j) Radar charts of key performance metrics of DCFs and DPCCFs. DCF, dye‐based composite filter; DPCCFs, dye/photonic crystal trans‐reflective color filters; PSU, polysulfone; TGA, Thermogravimetric Analysis.

Organic dyes typically suffer from shot lifespans, highlighting the importance of evaluating the stability of the fabricated DPCCFs through accelerated UV exposure and thermal tests. Under 24 h of UV irradiation at room temperature, the transmission and reflection spectra of the DPCCF remained almost unchanged (Figure 5b–d, Figure S12), and the structural color of DPCCF remained highly stable, showing a color difference Δ*E* < 0.20 before and after illumination (Table 1), whereas the reference DCF exhibits a slight decrease in transmittance from 90% to 86% (Figure S13). The enhanced UV stability is attributed to the CeO_2_@SiO_2_ nanospheres, which absorb UV light below 350 nm (Figure 5a). CeO_2_@SiO_2_ PCs shields the embedded dye molecules from UV exposure and reduces photo‐oxidative degradation, leading to improved long‐term optical durability of the filters.

**TABLE 1:** Coordinate values and Δ*E* of reflective color of DPCCF before and after irradiation.

Films	Before irradiation			After irradiation			Δ*E*
	L*	a*	b*	L*	a*	b*	
Y‐1 DPCCF	59.81	−16.07	−13.59	59.77	−16.15	−13.44	0.17
M‐1 DPCCF	69.77	−32.08	13.33	69.68	−32.13	13.39	0.12
C‐1 DPCCF	65.22	18.48	−5.66	65.21	18.38	−5.61	0.11

Abbreviation: DPCCF, dye/photonic crystal trans‐reflective color filters.

Thermal stability of DPCCFs was assessed by comparing the transmission spectra of DPCCFs before and after heat treatment at 230°C for 30 min. As shown in Figure 5f–h, the transmittance of the composite filters remains unchanged, confirming excellent thermal stability. The reflection spectra also remain unchanged in Figure S14, and the structural color appearance of DPCCF exhibited exceptional thermal stability, with a color difference Δ*E* < 0.30 before and after thermal treatment (Table 2). This stability is owing to the high decomposition temperatures of the dyes (Y‐1, M‐1, and C‐1) and PSU matrix (Figure 5e), which exceed 230°C. Additionally, PSU possesses high thermal decomposition (*T*_d_) and glass transition temperatures (*T*_g_) due to the strong intermolecular forces associated with its thermally stable aromatic backbone (Figure S15). The introduction of alkyl side chains in the dyes, along with robust π–π stacking interactions between the dye cores (benzene ring in Y‐1, perylene ring in M‐1, and phthalocyanine ring with conjugated groups in C‐1) and the aromatic segments in the PSU main chain, enhances the dye‐polymer compatibility mutually. This prevents dye aggregation, further improving the thermal stability of the composite filters.

**TABLE 2:** Coordinate values and Δ*E* of reflective color of DPCCF before and after baking at 230°C.

Films	Before baking			After baking			Δ*E*
	L*	a*	b*	L*	a*	b*	
Y‐1 DPCCF	58.72	−15.52	−13.23	58.77	−15.63	−13.46	0.26
M‐1 DPCCF	71.24	−34.3	14.18	71.12	−34.38	14.24	0.16
C‐1 DPCCF	64.78	18.57	−5.54	64.81	18.38	−5.56	0.19

Abbreviation: DPCCF, dye/photonic crystal trans‐reflective color filters.

A comparative performance assessment of DCFs and DPCCFs was conducted using hexagonal radar charts (Figure 5i,j). The plots quantify six key performance metrics on a normalized 0–100 scale: the FWHM of the transmission spectral band rejection (transmission FWHM), reflectance, CMY transmission color, RGB reflection color, UV stability, and thermal stability. The expansion of polygonal area in the radar charts quantify its multifunctional advantages, revealing the distinct performance hierarchies between DCFs and DPCCFs. In Figure 5i, although DCFs exhibit high CMY transmission color and excellent UV stability and thermal stability (up to 100%), it falls short in key performance metrics such as reflectance (0%), RGB reflection color (0%), and a relatively narrow transmission FWHM (∼60 nm), thereby underscoring its functional limitations. In sharp contrast, DPCCFs exhibit superior performance across all categories (Figure 5j). RGB reflection color, CMY transmission color, thermal and UV stability metrics of DPCCFs exceed a score of 90, while reflectance surpasses 70%, resulting in vibrant and high‐purity RGB reflection. DPCCFs also show a broader transmission FWHM (∼80 nm), indicating improved spectral definition and deep CMY color output. These results confirm that DPCCFs represent a comprehensive advancement over traditional DCFs, offering enhanced spectral precision, chromatic performance, and environmental robustness simultaneously—making them strong candidates for high‐performance optical applications.

### 2.6 Application of DPCCFs

**FIGURE 6:**
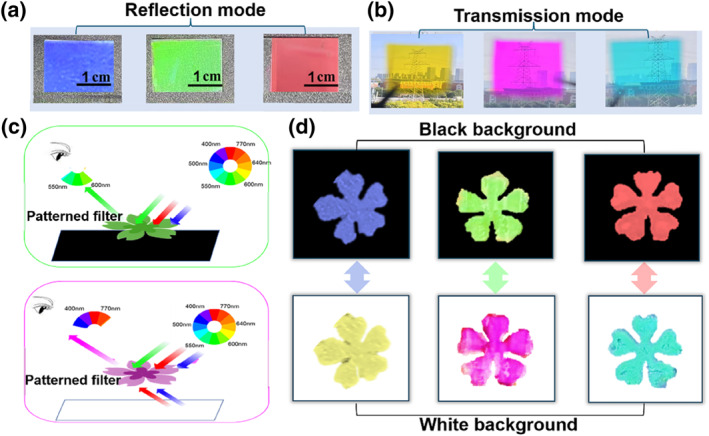
DPCCFs applied as transmission‐reflection color filters: (a) Photographic images of the DPCCFs that exhibit reflective RGB; (b) Photographic images of the DPCCFs that exhibit transmissive CMY; (c) Illustration of the color tuning mechanism for the DPCCFs on black and white backgrounds; (d) Photographs of DPCCFs with three different colors on black and white backgrounds. CMY, cyan, magenta, yellow; DPCCFs, dye/photonic crystal trans‐reflective color filters.

PCCFs were employed to dynamically modulate the object coloration through dual‐mode operation, demonstrating their versatility for advanced optical applications. As illustrated in Figure 6a, DPCCF films display vivid structural colors—blue, green, and red—in reflective mode, corresponding to their wavelength‐selective filtering capabilities. Figure 6b illustrates the broadband transmission and high optical transparency of the devices, with inset images revealing distinct yellow, magenta, and cyan chromatic outputs, confirming clear visibility and dual‐mode functionality. This dual‐mode functionality arises from the synergistic interaction between organic dyes and colloidal nanospheres, enabling precise spectral manipulation and highlighting their potential for optoelectronic device integration.

This dual‐mode functionality also enables DPCCFs to be applied in information storage and encryption. As depicted in Figure 6d, DPCCF‐based “flower” patterns display highly saturated trichromatic hues (blue, green, red) on black substrates, while exhibiting complementary colors (yellow, magenta, cyan) on white substrates. Furthermore, the optical modulation mechanism was exemplified by the M‐1 DPCCF (Figure 6c). On a black substrate, PCs selectively reflect green wavelengths via Bragg diffraction. The reflected light is subsequently filtered through dye absorption, producing a high‐purity green reflection, while non‐resonant red and blue wavelengths are completely absorbed by the black substrate (Figure 6c, top). When the substrate is changed to white, the green light remains reflected, while transmitted red and blue wavelengths are diffusely reflected from the white substrate. These reflected components undergo additive color mixing (red + blue = magenta), which synergizes with the intrinsic absorption profile of the dye, resulting in a deep magenta hue (Figure 6c, bottom). Similar substrate‐responsive color changes were observed in Y‐1 and C‐1 DPCCF “flowers,” confirming universal substrate‐responsive behavior. The reversible, substrate‐dependent chromatic response of DPCCFs makes them well‐suited for anti‐counterfeiting technologies and encrypted data storage. In summary, strategically engineered DPCCFs enable substrate‐agnostic multichromatic output, offering transformative potential for adaptive optical filters, high‐fidelity colorimetric printing, and advanced information encryption technologies.

## 3 CONCLUSIONS

In this work, we have developed a novel strategy for integrating organic dye‐functionalized PSU into PC structures to develop high‐performance trans‐reflective color filters. Notably, the maximum absorbance wavelength of the synthesized dye‐based PSU is precisely aligned with the maximum reflectance wavelength of the PCs. The refractive index‐matched PCs induce spectral broadening of the dye's transmission profile, enabling a synergistic photonic–dye interaction. This yields a transmission FWHM of approximately 80 nm—significantly wider than that of conventional single PCs (∼30 nm) and dye‐based filters (∼60 nm)—and enhances the purity of the transmission colors: yellow (0.410, 0.510), magenta (0.446, 0.231), and cyan (0.114, 0.481). Simultaneously, the selective absorption properties of the dyes not only enable their function as transmissive color filters but also suppress scattered light, thereby improving reflection color purity (FWHM = 25 nm). This leads to vivid reflection colors, such as blue (0.186, 0.635), green (0.190, 0.010), and red (0.712, 0.281). Furthermore, the filters exhibit exceptional light (Δ*T* < 1%) and thermal stability (Δ*T* < 2%). Overall, this work provides a robust and scalable framework for the design of advanced optical filters capable of dual‐mode operation in reflection and transmission. It offers a promising approach for fabricating stable micro‐ and nanoscale optical systems with enhanced color purity, light and thermal stability.

## 4 EXPERIMENT

### 4.1 Spectral simulation

Theoretical reflection spectra were simulated using the FEM (wave optics module in COMSOL Multiphysics) for distinct composites of homogeneous nanospheres and high‐RI core/low‐RI shell nanospheres. In calculation, face‐centered cubic (FCC) colloidal crystals were modeled with the (111) lattice in the *x*–*y* plane, and a plane perpendicular to the (111) plane was introduced along the *z*‐axis. The homogeneous nanospheres were identical in size as core–shell nanospheres, with average RI of core and shell parts calculated using Equation (1):

(1) $$n_{\text{average}}=n_{\text{core}}X_{\text{core}}+n_{\text{shell}}X_{\text{shell}}.$$

The core RI was set to 1.90 (represented by CeO_2_), and the shell RI to 1.45 (represented by SiO_2_). Using these parameters, two types of colloidal crystal structures were constructed with a core‐shell volume fraction, maintaining a total thickness of 26 μm (M‐1 PSU/PCs trans‐reflective color filters and PSU/PC trans‐reflective color filters). The packing fraction for both composite structures was fixed at 0.74, consistent with the FCC arrangement. Simulations were conducted over a wavelength range of 400–800 nm, with a 1 nm gap resolution.

### 4.2 Materials

Cerium (III) nitrate hexahydrate (Ce(NO_3_)_3_·6H_2_O, 99.5%) and propylene carbonate were obtained from Aladdin Co. Ltd. Polyvinyl pyrrolidone (PVP, *M*_w_ = 29,000), PSU (*M*_w_ = 80,000), and diethylene glycol (99%) were purchased from Sigma Aldrich. Ethylene glycol (EG, 99%) and ethanol (99.9%) were supplied by Tianjin Tianli Chemical Reagent Corporation. Deionized water was used in all experiments.

### 4.3 Synthesis of CeO_2_@SiO_2_ core‐shell colloidal nanospheres

CeO_2_ nanospheres were prepared via a high‐temperature polyol reaction using SiO_2_ nanospheres as seeds. The SiO_2_ seeds (20 nm) were synthesized through hydrolysis of tetraethyl orthosilicate (TEOS) (5.5 mL) in water (87 mL) with arginine (0.087 g) as surfactants. To synthesize CeO_2_ nanospheres with an average diameter of 155 nm, PVP (2.5 g), Ce(NO_3_)_3_·6H_2_O (5.2 g), and the SiO_2_ seed solution (250 μL) were dissolved in EG (20 mL) under magnetic stirring. The flask was first placed inside an oil bath at 65°C for 3 h, which was heated by a thermomagnetic stirrer. Then, the transparent solution was heated to 155°C under N_2_ atmosphere for 1 h, which gradually turned to a yellow slurry after the reaction temperature was reached. After the solution was cooled down to room temperature, CeO_2_ nanospheres were isolated by centrifugation, washed with ethanol for three times, and calcined at 500°C for 5 h. The CeO_2_ was prepared The sizes of CeO_2_ nanospheres (80–150 nm) were controlled by adjusting the dosage of Ce(NO_3_)_3_·6H_2_O (i.e., D CeO_2_∼80 nm at 5.2 g, D CeO_2_∼100 nm at 5.0 g, D CeO_2_∼120 nm at 4.8 g).^[38]^ The CeO_2_@SiO_2_ microspheres prepared according to the above steps consist of three different particle sizes: ∼175 nm (15 nm–99 nm–61 nm SiO_2_–CeO_2_–SiO_2_), ∼215 nm (15 nm–127 nm–73 nm SiO_2_–CeO_2_–SiO_2_), and ∼250 nm (15 nm–153 nm–82 nm SiO_2_–CeO_2_–SiO_2_). The comprehensive refractive indices of these spheres are ∼1.58, ∼1.59, and ∼1.59, respectively, which are closest to the refractive index of PSU (Δ*n* < 0.04). The reflection color from the PCs constructed using these three particles is blue, green, and red, respectively. Therefore, these three spheres were selected as the template building blocks for the subsequent construction of PC structures.

For the representative synthesis of CeO_2_@SiO_2_ core–shell nanospheres, 10 mL of a 2 wt% CeO_2_ nanospheres suspension was dispersed in a glass vial of 30 mL of ethanol with ammonia (9 mL) and deionized water (10 mL). The mixed solution was quickly stirred at 500 rpm at 50°C. To control the shell thickness, 2.5 mL of TEOS (99.999%, Sigma‐Aldrich) was dissolved in 20 mL of ethanol and added dropwise into the vial by an automatic syringe pump. After 1 h of Stöber reaction, the resulted CeO_2_@SiO_2_ core‐shell nanospheres were purified by repeated centrifugation and redispersion in ethanol.

### 4.4 Fabrication of DCFs

Composite filters were prepared using organic dye (0.0090 g) and PSU (1.000 g). Both were dissolved in PGMEA (10 g) to form the dye‐polymer matrix. Then, the solution was drop‐cast into a glass template, and dried in an oven at 75°C for 2 h to fully evaporate the solvent and cure the polymer, forming DCFs.

### 4.5 Fabrication of DPCCFs

Composite color filters were prepared using CeO_2_@SiO_2_ colloidal crystals and organic dye with maximum absorption wavelength located in the blue range of the PC band gap. CeO_2_@SiO_2_ colloidal nanospheres dispersed in ethanol were self‐assembled into colloidal crystals on a glass slide (76 × 26 mm) using the substrate via a convective self‐assembly method. The surface of the substrate was cleaned and then hydrophilized by oxygen plasma treatment. The substrate was immersed in a colloidal suspension of core–shell nanospheres. The vertical deposition process took 3 days on a bench at room temperature without any disturbance. During the evaporation of ethanol, the solution meniscus was moving downward, resulting in uniform colloidal crystal assembly on both sides of the substrate. The thickness of crystalline layers was controlled to 26 μm by adjusting the weight fraction of the CeO_2_@SiO_2_ nanosphere suspension in ethanol. PSU homopolymer was dissolved N,N‐Dimethylformamide (DMF), and a 0.90 wt% dye‐based polymer solution in Section 4.4 was drop‐cast into colloidal crystals. The sample was then fully evaporated in an oven at 75°C for 2 h, and the polymer was solidified, producing transparent composite PC filter.

### 4.6 Fabrication of PCCFs

PCCFs were prepared by convective assembly of monodisperse CeO_2_@SiO_2_ nanospheres on glass slides in ethanol. PSU (1.000 g) was dissolved in DMF (10 g), and the solution was drop‐cast into a densely arranged colloidal PC template. After drying in an oven at 75°C for 2 h, the solvent was completely evaporated and the polymer was cured to obtain transparent composite PC filters.

### 4.7 Characterization

The colors of photonic filters were observed under an optical microscope in reflection mode (Nikon, Eclipse 80i). Reflection spectra were measured by a fiber‐coupled spectrometer (Ocean Optics, Inc., BH‐2000‐BAL), and absolute transmittances of composite filters were recorded on an ultraviolet–visible (UV–Vis) spectrophotometer (4100). SEM (Hitachi S‐4300) and TEM (JEOL JEM‐2100F) were used to examine the morphology of core–shell colloidal nanospheres and the cross‐sectional images of photonic filters. X‐ray diffraction patterns of CeO_2_ nanospheres was recorded by Rigaku Ultima VI X‐ray diffractometer operated at 35 kV and 40 mA. Zeta potential of CeO_2_@SiO_2_ nanospheres was measured by Malvern Zetasizer Nano ZS90 in neutral solution at room temperature.

### 4.8 Transmission electron microscopy characterization

The dried microspheres uniformly were dispersed in anhydrous ethanol to prepare a 0.01 wt% dispersion. Deposit a droplet of this dispersion onto a copper mesh grid coated with an ultrathin carbon film, then allow it to dry naturally in a contaminant‐free environment for 12 h. For TEM characterization, analyses were performed using a Tecnai G220 S‐Twin instrument operated at 200 kV. This included bright‐field imaging, selected area electron diffraction, and high‐resolution TEM.

### 4.9 Thermal and UV stability tests

Thermal stability was assessed by comparing the transmittance of filters before and after heat treatment at 230°C. For UV stability, filters were irradiated with a 365 nm LED lamp for 5 min, and the optical density of the filters was set to 20 mW·cm^−2^ (The integrated energy is 16.7 Wh/m^2^) by adjusting the distance between the filters and the lamp, following GB/T 31370.2–2015 standards. Transmission spectra of the filters before and after irradiation were measured using a high‐precision color analyzer. Thermal and UV stability of color filters were calculated using Equations (2) and (3), respectively:

(2) $$\text{Thermal}\;\text{stability}=\left(T_{0}-T_{1}\right)/T_{0},$$

(3) $$\text{UV}\;\text{stability}=\left(T_{2}-T_{3}\right)/T_{2},$$

where *T*_0_, *T*_2_ are the transmittance values before heat treatment or irradiation, and *T*_1_, *T*_3_ are the transmittance values after heat treatment or irradiation, respectively.

In the CIE chromaticity diagram (CIE 1931), excitation purity quantifies a color's saturation relative to a reference white point (like D65). The formula is

(4) $$\text{Purity}=\frac{\sqrt{{\;\left(x-x_{n}\right)}^{2}+{\;\left(y-y_{n}\right)}^{2}}}{\sqrt{{\;\left(x_{d}-x_{n}\right)}^{2}+{\;\left(y_{d}-y_{n}\right)}^{2}}},$$

where (*x*, *y*): Chromaticity coordinates of the test color. (*x*_*n*_, *y*_*n*_): Chromaticity coordinates of the white point (D65: *x*_*n*_ = 0.3127, *y*_*n*_ = 0.3290). (*x*_*d*_, *y*_*d*_): Chromaticity coordinates of the dominant wavelength on the spectral locus (the point where the line from the white point through the test color intersects the spectrum boundary).

### 4.10 Reflectance and transmittance spectroscopy tests

Reflectance spectra were measured using a Hitachi U‐4100 spectrophotometer over the 400–780 nm wavelength range, with a fixed 5° angle between the incident light and detector. In contrast, transmission spectra of blank films and CeO_2_@SiO_2_ composite films were acquired in the integrating sphere mode (Hitachi U‐4100, Japan) at 0° incidence angle.

## CONFLICT OF INTEREST STATEMENT

The authors declare no conflicts of interest.

## ETHICS STATEMENT

No animal or human experiments were involved in this study.

## Supporting information

Supporting Information S1

## Data Availability

The data that support the findings of this study are available from the corresponding author upon reasonable request.
