# Supplementary material for: Trans‐reflective color filters with brilliant colors by integrating organic dyes with photonic crystals
Source: Smart Mol. 2026 Jan 30;4(1):e70037. doi: 10.1002/smo2.70037 (PMC13104109; doi:10.1002/smo2.70037)
Supplement: Supplementary file 1 — Supporting Information S1 [file SMO2-4-e70037-s001.docx]

Supporting Information

Title: Trans-Reflective Color Filters with Brilliant Colors by Integrating Organic Dyes with Photonic Crystals

Shi Li, Yong Qi, Wenbin Niu, Suli Wu, Bingtao Tang, Wei Ma, Shufen Zhang*

The key characteristics of the CeO_2_@SiO_2_ nanospheres are depicted in Figures S1a–1f. Notably, the XRD spectra of the CeO_2_ nanospheres closely match the reference data (JCPDS 81-0792), confirming the successful synthesis of CeO_2_. Additionally, the peak observed at 77.26°, corresponding to the silica phase, verifies that CeO_2_ was effectively encapsulated by SiO_2_. (Figure S1b) The FTIR spectra of CeO_2_ and CeO_2_@SiO_2_ are presented in Figure S1c. Peaks at 663 cm⁻¹ and 562 cm⁻¹ indicate the characteristic vibrational modes of CeO_2_, while absorption bands at 1074 cm⁻¹, 798 cm⁻¹, and 454 cm⁻¹ are attributed to Si–O bonding. The peak at 948 cm⁻¹, associated with the Si–O–Ce vibrational band, further confirms the successful synthesis of CeO_2_@SiO_2_ nanospheres. The size distribution of the CeO_2_@SiO_2_ nanospheres, shown in Figure S1d, demonstrates a polydispersity index (PDI) of less than 0.2, indicating a uniform distribution of nanosphere sizes. This uniformity suggests that the CeO_2_@SiO_2_ nanospheres are highly suitable for constructing photonic crystal structures.


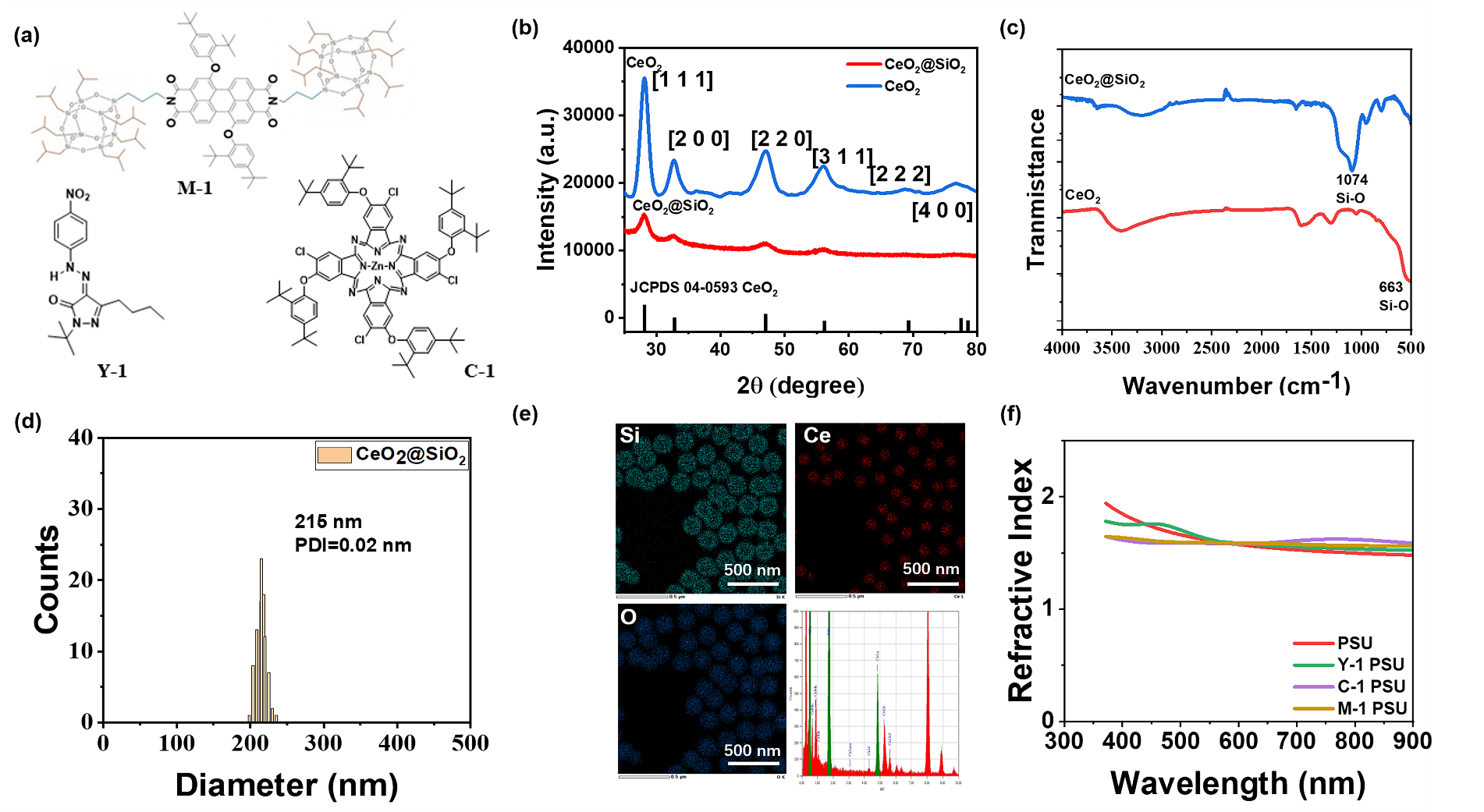


Figure S1 (a) Molecular formulae of three organic dyes. (b) XRD spectra of CeO_2_ and CeO_2_@SiO_2_ nanospheres. (c) FTIR analysis of CeO_2_ and CeO_2_@SiO_2_ nanospheres. (d) PDI of CeO_2_@SiO_2_ nanospheres. (e) EDS of CeO_2_@SiO_2_ (f) Refractive index (n) of PSU and three organic dyes (Y-1, M-1, C-1) in PSU.


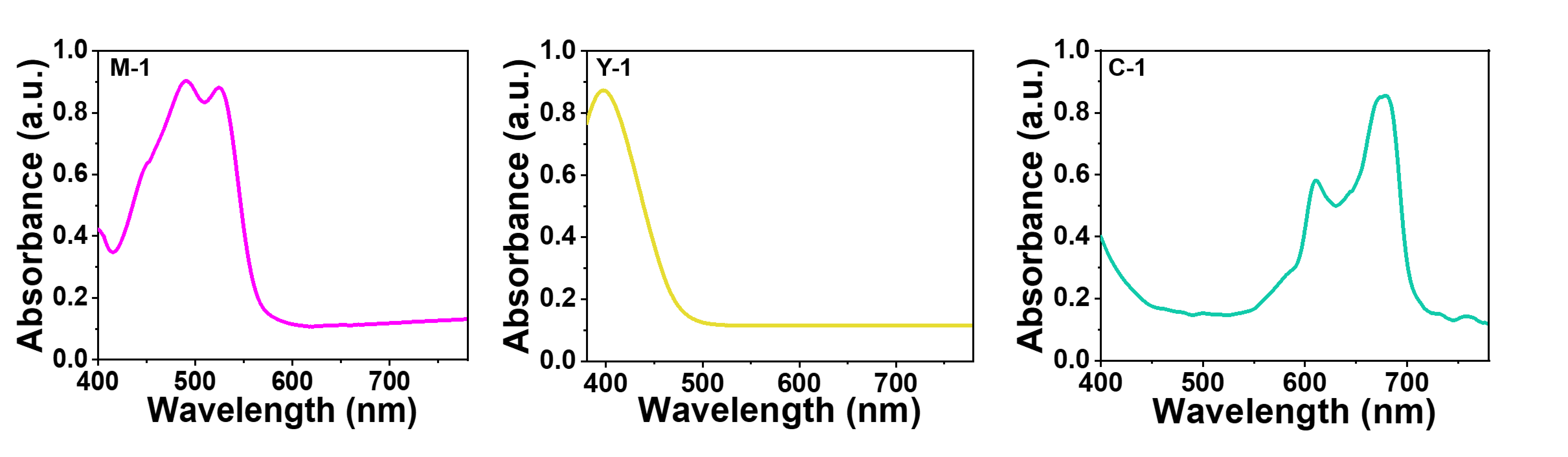


Figure S2 Absorbance spectra of three organic dyes (Y-1, M-1, C-1) in PGMEA.


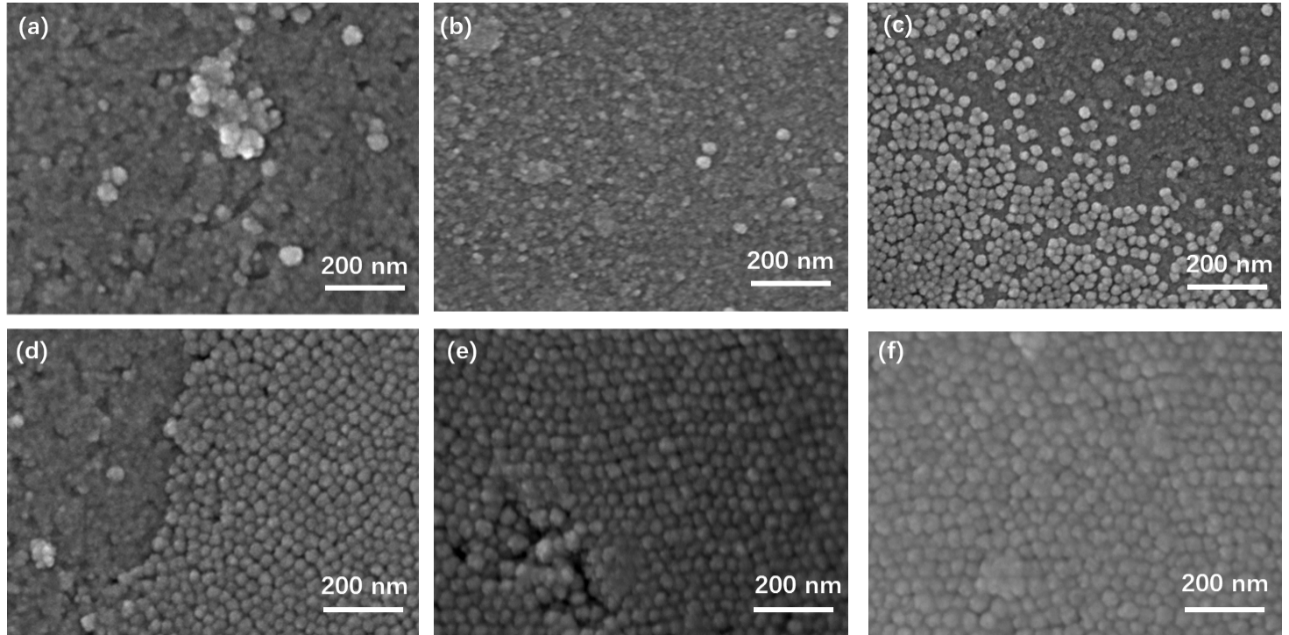


Figure S3. SEM images of core SiO2 microspheres


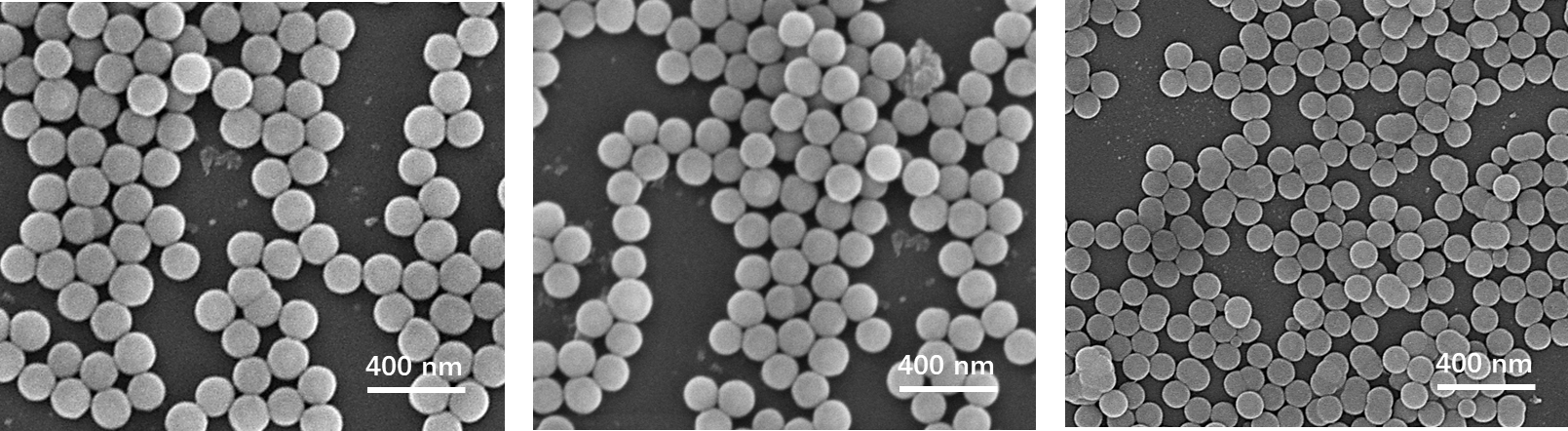


Figure S4 SEM images of CeO_2_ microspheres with different diameters (155 nm, 136 nm, 110 nm, from left to right).


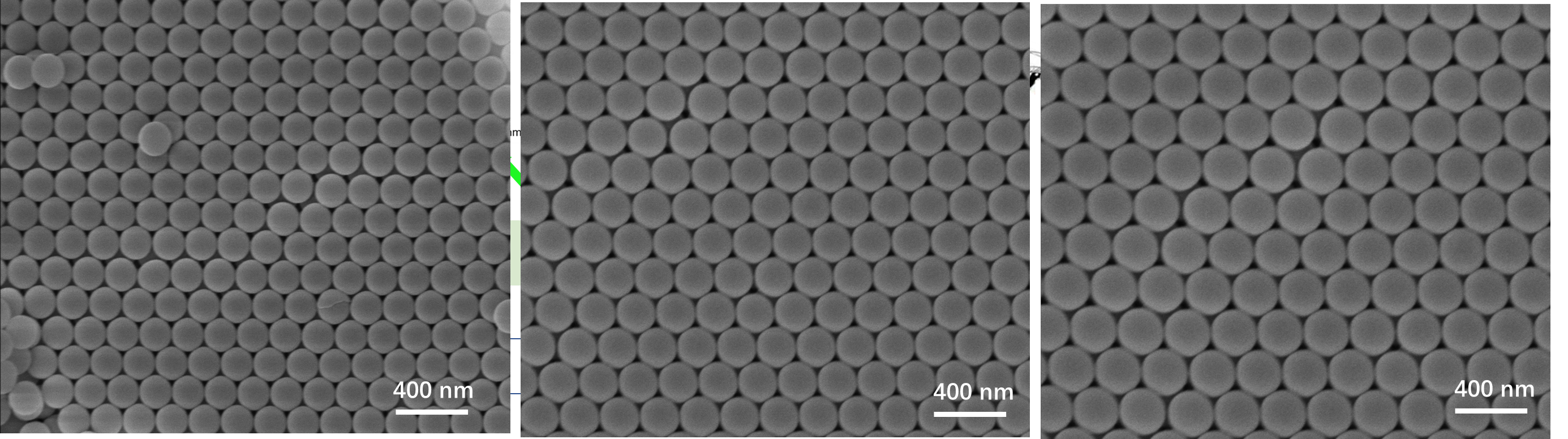


Figure S5 SEM images of CeO_2_@SiO_2_ microspheres with different diameters.


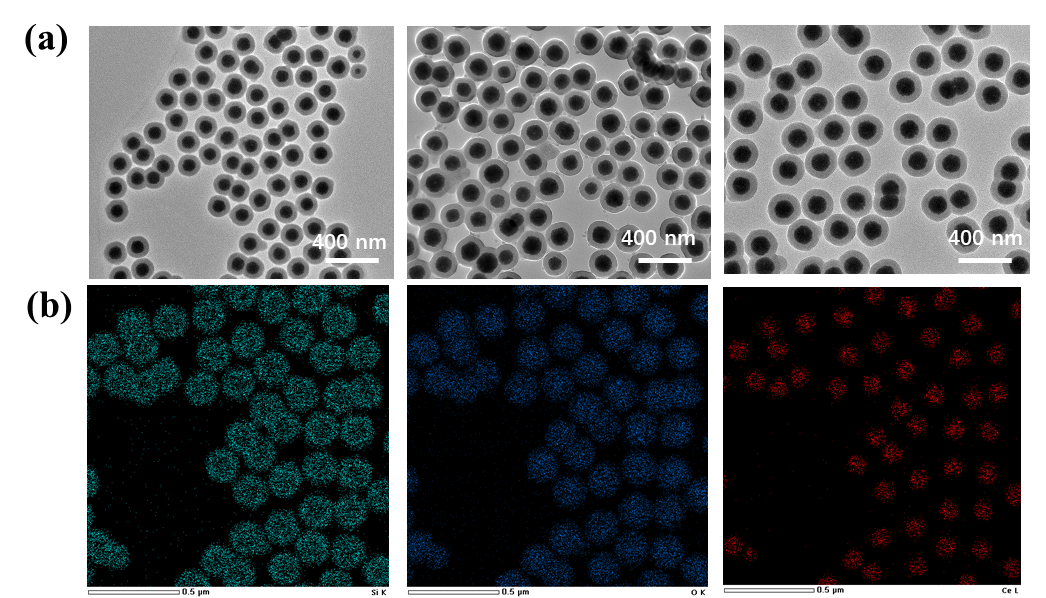


Figure S6 (a)TEM images of CeO_2_@SiO_2_; (b) EDS of CeO_2_@SiO_2_ with diameter at 215 nm.


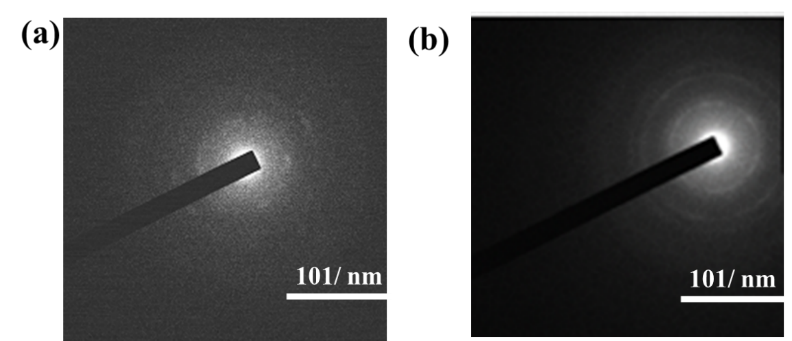


Figure S7 TEM images of CeO_2_ microspheres.

|  |  |
| --- | --- |
|  |  |
|  |  |
|  |  |
|  |  |

Figure S8 DLS of CeO_2_ and CeO_2_@SiO_2_.

Table S1 Zeta potential of Ce(HCOO)_3_, CeO_2_ and CeO_2_@SiO_2_.

| Colloidal particles | T(℃) | Zeta Potential（mV） |
| --- | --- | --- |
| $\text{Ce}(HCOO$)_3_ | 25 | 36.6 |
| CeO_2_ | 25 | 12.2 |
| CeO_2_@SiO_2_ | 25 | -44.9 |


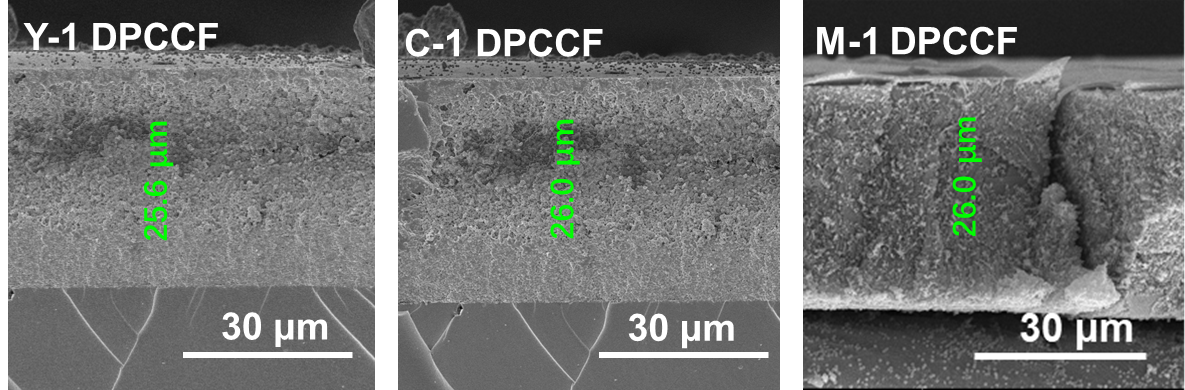


Figure S9 Cross-sectional SEM images of green composite opal films with different thicknesses of ordered layers.


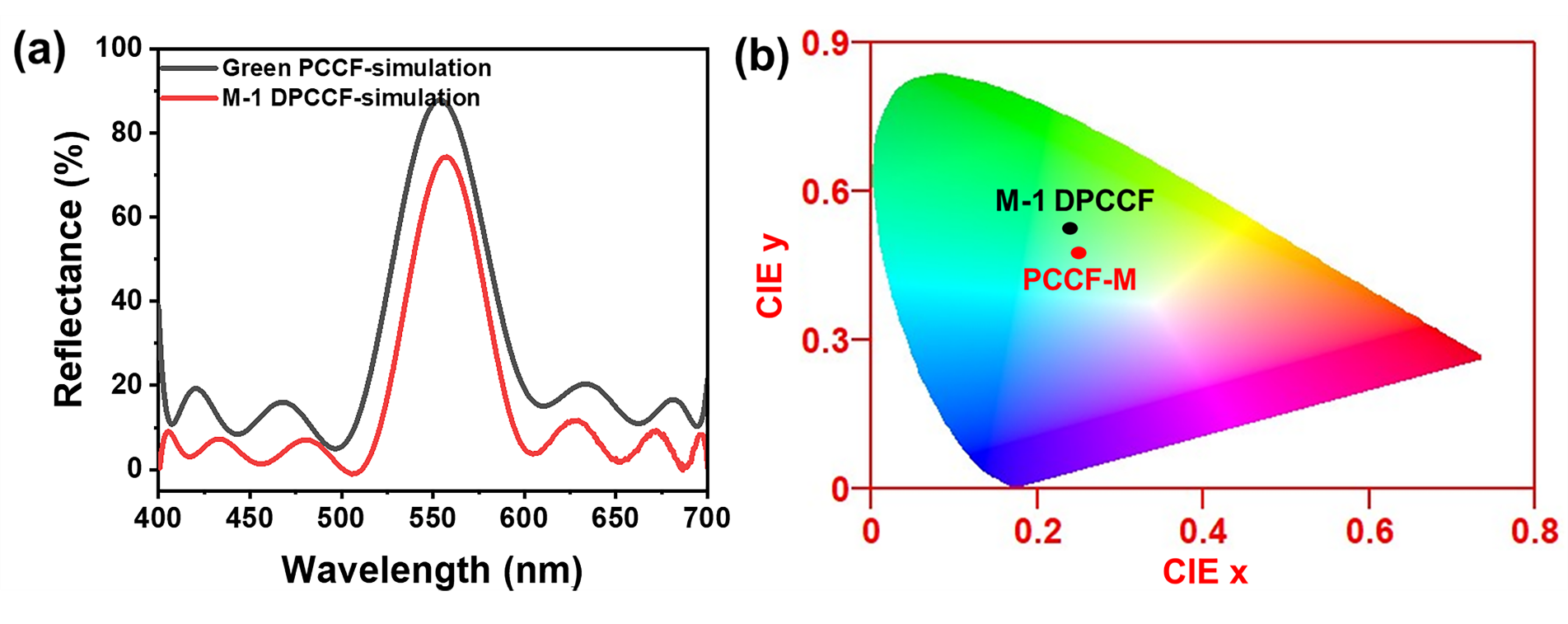


Figure S10 Simulated and experimental results of the optical properties of the M-1 DCF, PCCF-M, and M-1 DPCCF: (a) simulated reflection spectra of the PCCF-M and M-1 DPCCF; (b) corresponding color coordinates on the CIE 1931 chromaticity diagram calculated from all the reflection spectra presented in (a)


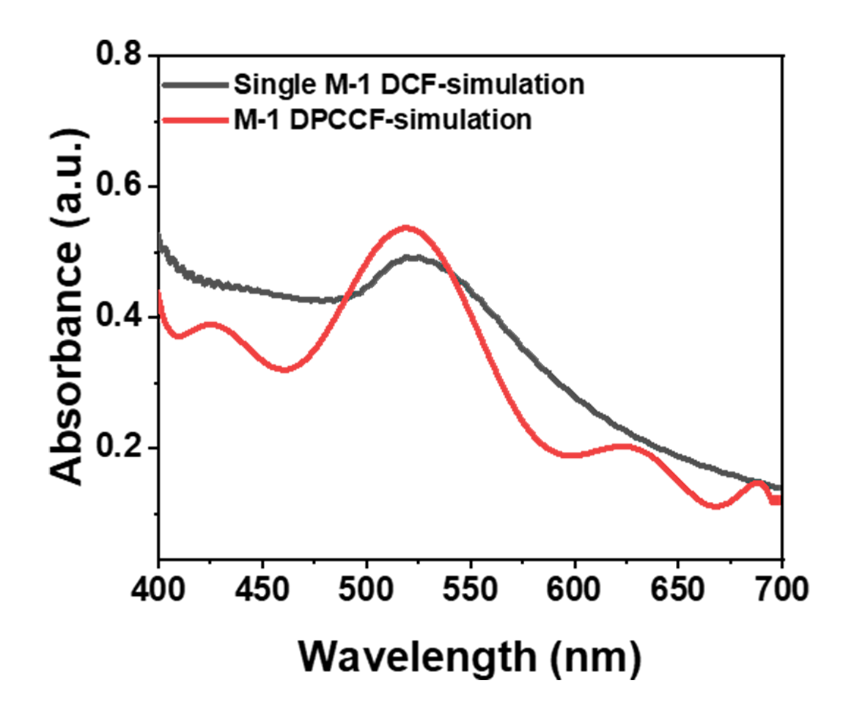


Figure S11 Simulated absorption spectra of M-1 DCF and M-1 DPCCF employing same mass fraction of dye M-1


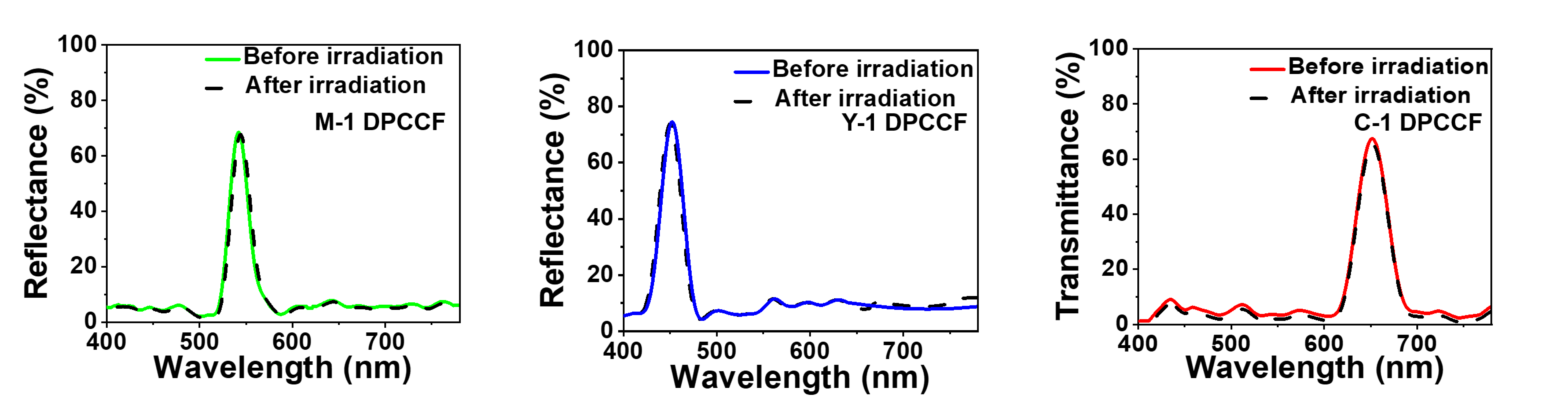


Figure S12 Reflection spectra of three films (Y-1 DPCCF, M-1 DPCCF, C-1 DPCCF) before and after irradiation.


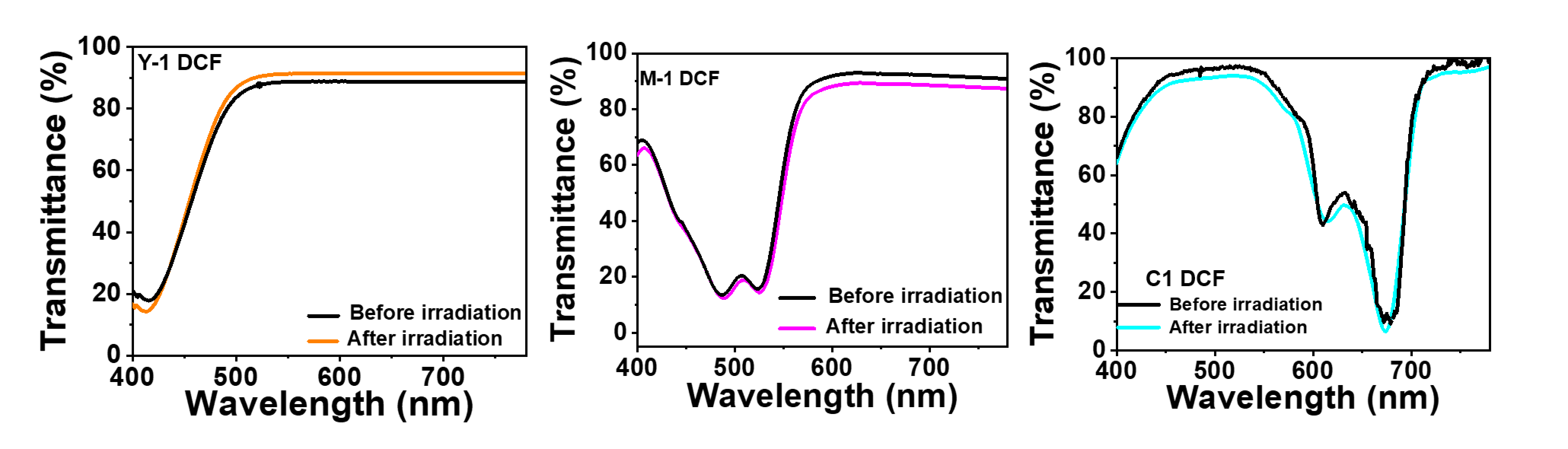


Figure S13 Transmission spectra of Y-1 DCF, M-1 DCF, and C-1 DCF before and after irradiation.


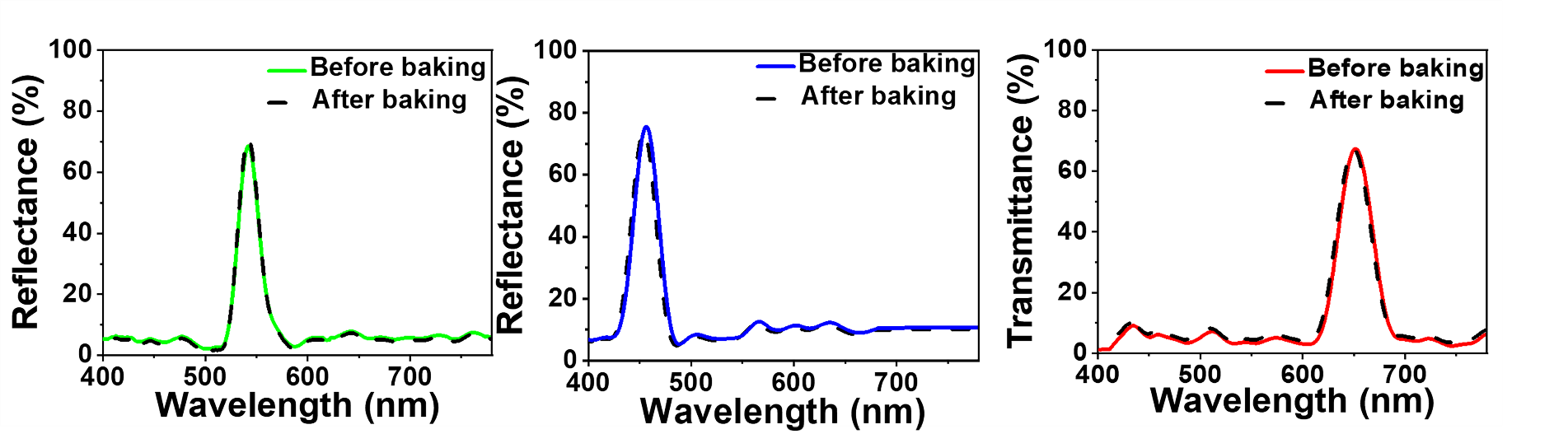


Figure S14 Reflection spectra of three films (Y-1 DPCCF, M-1 DPCCF, C-1 DPCCF) before and after baking.


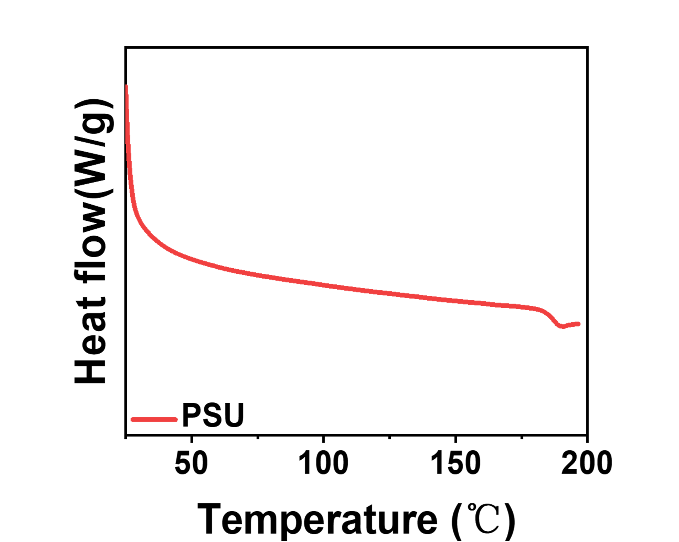


Figure S15 DSC curves of polymeric binders PSU.

Finite-difference time-domain (FDTD) simulations were employed to model the electric field magnitude distributions within various thin-film structures across the visible spectrum (400 nm, 500 nm, 515 nm, 530 nm, 585 nm, 600 nm, and 700 nm). As indicated by the scale in Figure S16, the color mapping represents electric field strength, which correlates directly with photon energy density.

In pure polysulfone (PSU) films (Figure S16a), which exhibit no significant reflection, energy bands vary across 400–700 nm. However, the maximum field strength corresponds only to yellow-level energy, reaching approximately 0.82 [E/E₀].

For the M-1 PSU composite (Figure S16b), which exhibits maximum absorption at 530 nm with broadband visible absorption, regions of stronger light absorption display blue-shifted hues in the field map, corresponding to lower field strengths near 0.30 [E/E₀]. Notably, at 530 nm—the peak absorption wavelength—absorption by M-1 dye causes a progressive color shift from yellow to cyan (top to bottom), accompanied by a field strength reduction of ~0.26 [E/E₀]. At longer wavelengths (≥650 nm), where absorption weakens, field strengths increase significantly to ~0.80 [E/E₀].

Within the SiO₂-PSU structure (Figure S16c), diffuse reflection from SiO₂ microspheres generates distinct field patterns: At 400 nm, regions of diffuse reflection exhibit high-energy orange-red hues, corresponding to a field strength of ~0.85 [E/E₀]. At 530 nm (the reflectance maximum), intense surface reflection produces prominent orange-red coloring with a field strength of ~0.88 [E/E₀]. At 700 nm, weak diffuse reflection results in faint orange-red coloring near microsphere surfaces, with a field strength of ~0.83 [E/E₀].

In the reflectance spectrum of PCCF-M (SiO₂@CeO₂@SiO₂-PSU photonic crystal film; Figure S16d), the peak reflection occurs at 550 nm, with diffuse reflection observed across other visible wavelengths. Corresponding electric field distributions reveal: In regions of strong diffuse reflection (>585 nm), microsphere surfaces exhibit high-energy red hues with field strength ≈1.00 [E/E₀]. In weak diffuse reflection regions (<515 nm), microsphere surfaces display high-energy orange-red coloring with field strength ≈0.88 [E/E₀]. At 530 nm (proximal to the reflectance maximum), intense red coloring appears with uniform vertical energy distribution (field strength: 1.08 [E/E₀]), attributable to the absence of absorption in PCCF-M.

M-1 DPCCF (SiO₂@CeO₂@SiO₂-M-1-PSU) exhibits analogous behavior (Figure S16e), with peak reflection at 550 nm and broadband diffuse reflection: Strong diffuse reflection regions (>585 nm): Microsphere surfaces show high-energy red (field strength ≈0.97 [E/E₀]). Weak diffuse reflection regions (<515 nm): Microsphere surfaces exhibit high-energy orange-red (field strength ≈0.83 [E/E₀]). At 530 nm: Microsphere surfaces display red coloring (field strength ≈1.03 [E/E₀]). Compared to PCCF-M, M-1 DPCCF exhibits marginally reduced field strengths across wavelengths due to energy dissipation by M-1 dye absorption. Crucially, at 530 nm, M-1 DPCCF shows a vertical energy gradient transitioning from red to green (field strength declining to 0.72 [E/E₀]), corresponding to progressive dye absorption through the structure. This demonstrates that integrating dye molecules with photonic crystals reduces reflectivity while enhancing optical transmittance.


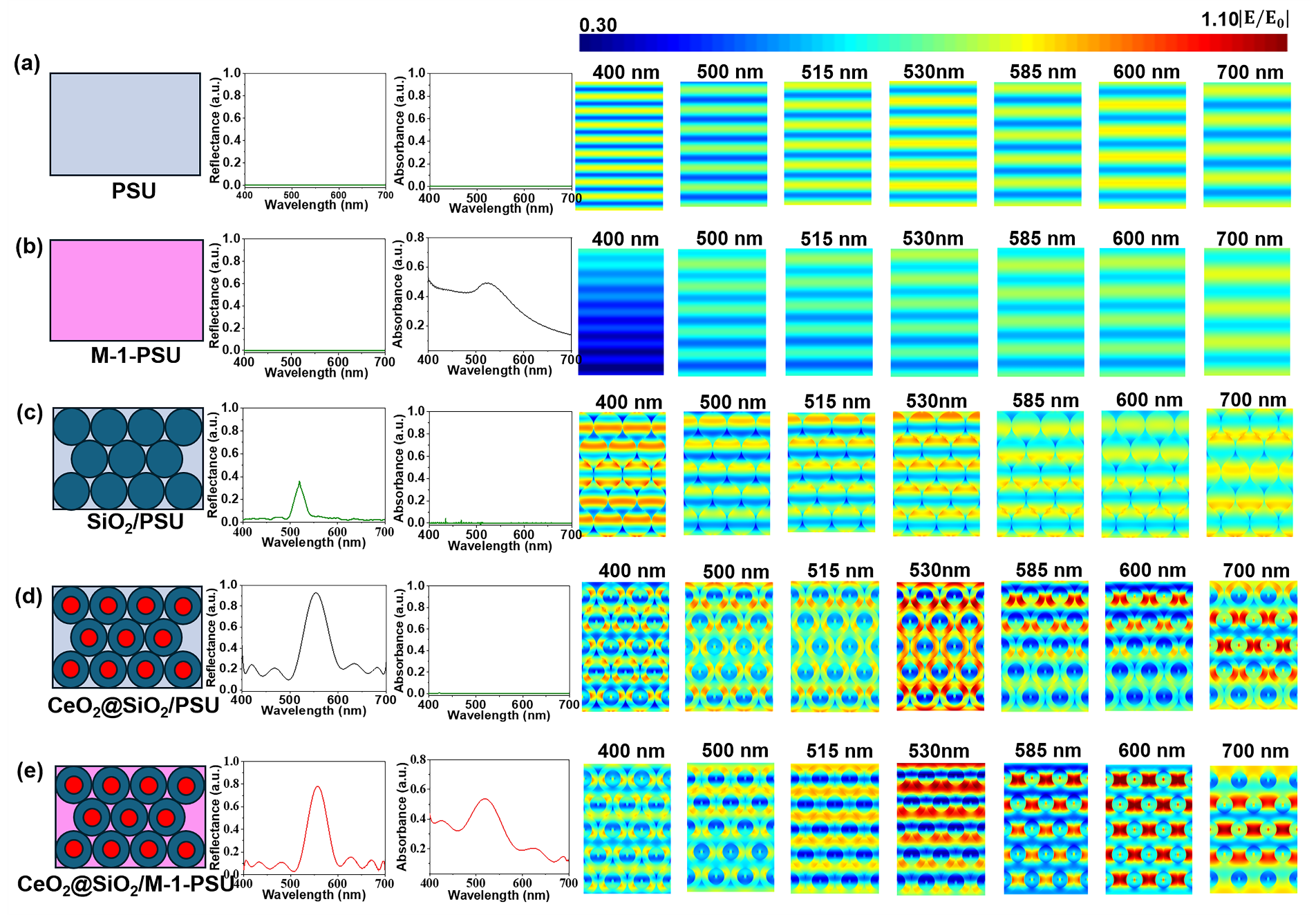


Figure S16 Structural diagrams, reflection spectrum, absorption spectrum, and the electric field distribution (400 nm, 500 nm, 515 nm, 530 nm, 585 nm, 600 nm and 700 nm) of the internal structure of films: (a) PSU; (b) M-1 DCF; (c) SiO_2_-PSU; (d) PCCF-M; (e) M-1 DPCCF
